# The impact of human immunodeficiency virus (HIV) service scale-up on mechanisms of accountability in Zambian primary health centres: a case-based health systems analysis

**DOI:** 10.1186/s12913-015-0703-9

**Published:** 2015-02-18

**Authors:** Stephanie M Topp, Jim Black, Martha Morrow, Julien M Chipukuma, Wim Van Damme

**Affiliations:** 1 Schools of Public Health and Medicine, University of Alabama, Birmingham, USA; 2 Centre for Infectious Disease Research in Zambia, PO Box 30338, Lusaka, Zambia; 3 Nossal Institute for Global Health, University of Melbourne, Level 4, 161 Barry Street, Alan Gilbert Building, Carlton, 3010 VIC Australia; 4 University of Lusaka, Plot No 37413, Mass Media, Lusaka, 101010 Zambia; 5 Public Health and Health Policy Unit, ITM-Antwerp, Sint-Rochusstraat 2, 2000 Antwerpen, Belgium; 6 School of Public Health, University of the Western Cape, Robert Sobukwe Road, Bellville, 7535 Republic of South Africa

**Keywords:** Accountability, HIV service scale-up, Primary health centres, Health systems

## Abstract

**Background:**

Questions about the impact of large donor-funded HIV interventions on low- and middle-income countries’ health systems have been the subject of a number of expert commentaries, but comparatively few empirical research studies. Aimed at addressing a particular evidence gap vis-à-vis the influence of HIV service scale-up on micro-level health systems, this article examines the impact of HIV scale-up on mechanisms of accountability in Zambian primary health facilities.

**Methods:**

Guided by the *Mechanisms of Effect* framework and Brinkerhoff’s work on accountability, we conducted an in-depth multi-case study to examine how HIV services influenced mechanisms of administrative and social accountability in four Zambian primary health centres. Sites were selected for established (over 3 yrs) antiretroviral therapy (ART) services and urban, peri-urban and rural characteristics. Case data included provider interviews (60); patient interviews (180); direct observation of facility operations (2 wks/centre) and key informant interviews (14).

**Results:**

Resource-intensive investment in HIV services contributed to some early gains in administrative answerability within the four ART departments, helping to establish the material capabilities necessary to deliver and monitor service delivery. Simultaneous investment in external supervision and professional development helped to promote transparency around individual and team performance and also strengthened positive work norms in the ART departments. In the wider health centres, however, mechanisms of administrative accountability remained weak, hindered by poor data collection and under capacitated leadership. Substantive gains in social accountability were also elusive as HIV scale-up did little to address deeply rooted information and power asymmetries in the wider facilities.

**Conclusions:**

Short terms gains in primary-level service accountability may arise from investment in health system hardware. However, sustained improvements in service quality and responsiveness arising from genuine improvements in social and administrative accountability require greater understanding of, and investment in changing, the power relations, work norms, leadership and disciplinary mechanisms that shape these micro-level health systems.

## Background

Between 1996 and 2008 global funding for HIV increased from US$300 million to an estimated US$15.6 billion [1]. A large proportion of this funding was directed towards a handful of countries in sub-Saharan Africa where HIV/AIDS was a major health, social and economic threat [2]. Justified in part by the emergency status of the epidemic, the exceptional levels of funding and rapidity of treatment services scale-up nonetheless spurred debate regarding the impact of disease-specific programs on recipient countries’ health systems [2-10]. Central to this debate was the question of whether such intense focus on a single disease could be harmful to the overall development of health systems in low- and middle-income settings.

Questions about the functions of, and interactions between, donor-funded HIV interventions and national health systems have been the subject of a number of expert commentaries, but comparatively few empirical research studies [9]. Amongst those studies that have focused on these issues, moreover, [11-14], critics have noted a bias towards national-level assessments, as well as a tendency to focus on measurement of effect. Far less research has been conducted to examine the *mechanisms* of effect (where found), that is, *how* and *why* HIV scale-up impacted on local health systems [9,15,16], although there are some notable exceptions [17-21]. The lack of empiric evidence in relation to these issues is concerning given the continued scale-up of antiretroviral therapy (ART) and the transformation of HIV into a chronic disease, which are placing increasing pressure on weak primary level health services [22,23].

The study reported here formed part of a larger research project that aimed to address the evidence gap vis-à-vis the influence of HIV service scale-up on Zambian primary health centres. An initial objective of the larger study was to produce theoretically informed insights relating to the mechanisms driving health centre performance. Previously reported findings pertaining to this objective [24] point to the critical role of mechanisms of accountability, amongst others (e.g. mechanisms of trust), in determining primary-level service quality and responsiveness.

The explicit focus of this article is to examine whether and how the establishment and scale-up of HIV services influenced mechanisms of accountability within the primary service domain, and, as a result, service quality and responsiveness. We then apply these findings to a consideration of whether there is merit in attempting to design disease-specific interventions that reflect the complexity in primary-level services, and, in the process, enable a more contextually comprehensive approach to the design and implementation of health system strengthening interventions.

### Study setting

The national HIV care and treatment program of the Government of the Republic of Zambia (GRZ) progressed in several distinct stages from the early 2000s. Between 2001–2003 a hospital-based HIV treatment program financed by government and patient co-payments was piloted [25]. In 2003, a radical policy shift introduced by then President Levy Mwanawasa promised free and universal access to ART, primarily supported by the (United States government-funded) President’s Emergency Plan for AIDS Relief (PEPFAR) and the (multi-lateral) Global Fund to Fight AIDS, Tuberculosis and Malaria (GFATM) [26]. As in many sub-Saharan nations with generalized epidemics, the early emphasis of HIV service rollout in Zambia was now placed on speedy implementation, justifying (at least initially) the bypassing of what some perceived to be weak or inefficient systems [25]. Between 2003 and 2010, scale-up was overseen by the Ministry of Health (MOH) but executed primarily by PEPFAR implementing partners, namely, non-government sub-contractors tasked with establishing and supporting (both materially and technically) HIV treatment services in government primary health facilities [27]. During this period, HIV services expanded from just two hospital-based treatment centres to encompass prevention of mother to child transmission (PMTCT) in some 1450 government primary health centres and health posts, and full ART services in over 150 health centres [28].

In 2010, PEPFAR implementing partners everywhere came under pressure from prime grantees and US agencies to harmonize the ‘vertical’ HIV services with the structures of the local health system [29]. In Zambia, as a result, direct salary support in the form of the ‘over time’ or ‘part time’ payments for providers working in ‘ART clinics’ was phased out and the money reallocated to capacity building activities such as additional clinical training and training of future trainers [25]. From 2011, as part of the same process, local non-government implementing partners were required to transition responsibility for some support activities (e.g. quality assurance checks, administrative support roles) to District and Provincial health offices. Funds previously channelled to these partners were now redirected to government institutions taking a greater role in (non-wage) support and technical assistance. According to officials from partner agencies and the Ministry of Health, this transition received considerable attention during the central planning stages, but was poorly managed on the ground^a^ [25,30,31]. The period of 2011–2012 saw a marked decline in direct material and technical support to primary level health centres, particularly in the large urban health centres that were each supporting HIV patient loads of between 3000 and 15,000 individuals.

### Study design

#### Conceptual framework

**Figure 1:**
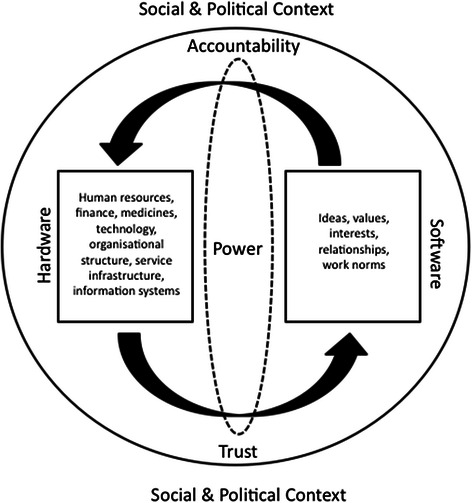
***Mechanisms of effect:*** **a framework for micro health systems analysis.**

This study and the larger project are situated in the field of health policy and systems research (HPSR) and were guided by insights from an emerging body of literature on the social and adaptive nature of health systems [32,33] and literature on micro-health systems of front-line health services [17,34-36]. In particular this study was guided by the *Mechanisms of Effect* framework (see [24]), which extended earlier theories by Sheikh *et al.* [37] suggesting that health system performance is a product of interactions between system ‘hardware’ (tangible components such as infrastructure, drugs, and human resources) and system ‘software’ (intangible components such as human values, power dynamics and norms). The adaptation posits (a) that the quality and responsiveness of front-line services are largely determined by mechanisms of accountability and trust, and (b) that it is these mechanisms that are the product of hardware-software interactions (see Figure 1). Recognising that social adaptive systems are shaped in part by feedback loops, this framework further suggests that accountability and trust (or their absence) may also become defining properties of the system as a whole.^b^

For this study we sought to refine our exploration of the impact of HIV services on accountability^c^ and so conducted a scoping review of health services and accountability literature to identify key themes of focus. The review suggested that *administrative* and *social* accountability were of most relevance for explorations of front-line, clinic-based health services [38-43]. Administrative accountability (elsewhere called bureaucratic or political accountability) is defined as healthcare providers’ sense of accountability to their managers in relation to clinical guidelines and pre-defined performance standards. Social accountability is defined as providers’ accountability to their clients (and broader community) in relation to the needs and expectations of these groups.

Our examination of social and administrative accountability was informed by Chandler and Plano’s definition of accountability as ‘*a condition in which individuals who exercise power are constrained by external means and internal norms’* [44]. This definition is significant in a number of respects. First, it points out that accountability is relevant to all individuals who exercise power, not only those who occupy formal positions of authority or those exercising legal/rational power. In the micro-health system setting this suggests that all actors (not just health centre managers or doctors) must be accountable. Second, this definition highlights the fact that accountability is not only achieved through ‘external means’ – indicating policies, rules, guidelines and other formal means – but also through ‘internal norms’ and self-policing. In the health centre setting, these internal norms might include perceptions of responsibility for delivering high quality services, a sense of obligation to the patients, or a strong work ethic. Third, the definition suggests (implicitly) that both external means *and* internal norms are necessary to generate accountability and that the constraint of power via only one or the other is likely to be insufficient.

In developing a typology of accountability we additionally drew on Brinkerhoff’s characterisation of accountability [39,45] as the product of effective mechanisms of *answerability* and of *enforceability.* Answerability implies the ability of a supervisor, official or client to obtain information about what is happening and why it is happening in a certain way. Enforceability refers to the ability of a supervisor official or client(s) to demand certain formal or informal standards be met and to seek redress if they are not.

**Figure 2:**
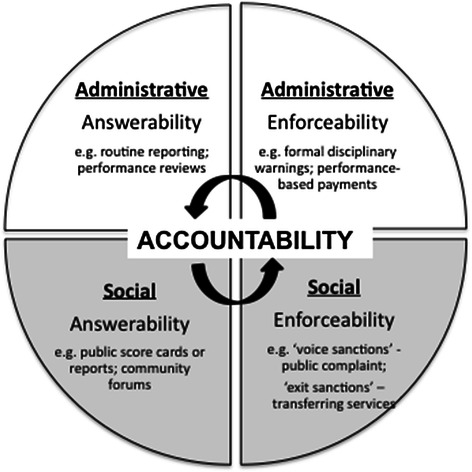
**Mechanisms of administrative and social accountability in a health centre setting.**

Figure 2 provides the typology of accountability adopted in this study, illustrating the domains of administrative and social accountability and examples of mechanisms of answerability and enforceability (representing both external means and internal norms) common to primary level services in low and middle income countries (LMIC).

## Methods

The overall aim of the study reported here was to understand whether and how the establishment and scale-up of HIV services influenced mechanisms of accountability. Specific objectives that guided our exploration included: to describe how the introduction of HIV care and treatment services influenced the organisation of resources and actors in each health centre; to describe whether, and in what ways, health centre components interacted to produce differing levels of accountability; and to explore the ways in which introduction of HIV care and treatment services influenced the interaction between system components, and, subsequently, the production of accountability (social or administrative) in this setting.

A full description of the study design and data collection methods used in this and the larger research project has been published previously [24]. Briefly, we adopted a multi-case study design using a theoretical replication strategy [45]. Case ‘units’ – four primary health centres located in two adjacent Districts, one urban one rural – were selected by the lead investigator (SMT) in consultation with District Medical Officers, and based on both empiric and anecdotal evidence of characteristics that enabled exploration of patterns of service delivery. Such characteristics included: average patient attendance data; vaccination coverage rates; and District officers’ descriptions of health centre performance. Data were collected between June and December 2011. Methods used at each case site included: in-depth interviews with a proportionate sample of healthcare workers from various levels (n = 60); semi-structured interviews with a quasi-random sample of patients (n = 180); review of health centre paper-based registers; and direct observation of facility operations spanning at least two weeks in each centre. Observation was both structured and semi-structured, and included the National Healthcare Standards Assessment tool for Zambian Health Facilities, and note-taking to record informal discussions and interactions during the period of residence. In addition, key informant interviews were held with government and non-government officials (n = 14) with specific knowledge or experience of the processes of HIV service scale-up.

Data for this study are primarily drawn from interviews with providers and patients and direct observations in the four health centres, although interviews with key informants including District officials and NGO were also used. During all interviews (patient, provider and key informants), respondents were asked a range of questions exploring the formal and informal relationships, processes and structures that influenced their own and others’ decision making and actions in the health centre. Analysis was carried out in three phases. Phase one, conducted concurrently with data collection, comprised collated notes and summaries of evidence for each health centre. In phase two, transcribed interviews were imported into NVivo QSR™ for electronic coding, and data were organised to produce a case description for each health centre [46] built around four major themes. These were: i) providers’ role in the health centre, their typical routine, and their position in relation to others in the facility; ii) challenges faced in day-to-day work; iii) perceptions of the work patterns and work culture in the facility, including relationships with colleagues and health centre managers; and iv) understanding of, and attitudes towards, the introduction of HIV services. Phase three involved a subsequent round of coding which focused on identifying how the different domains (administrative and social) and mechanisms (answerability and enforceability) of accountability were influenced by the new HIV services in each and across the four facilities. Deductive analysis was guided by codes developed from the Mechanisms of Effect (Figure 1) and Accountability (Figure 2) frameworks respectively, while inductive coding was used to incorporate new or emergent themes. Coded text and anonymised sources were collated in a word-processor document and printed, after which we synthesised evidence of the impact of HIV service establishment on mechanisms of social and administrative accountability. Negative case analysis was conducted through the identification of experiences or interactions that appeared to contradict our theoretical assumptions.

Written informed consent was obtained from all respondents for formal observations or interviews. The study received ethical clearance from the Human Research Ethics Committee (HREC) of the University of Melbourne (REF#:1035194) and the University of Zambia Biomedical Research Ethics Committee (REF#: 004-03-011).

## Results

Findings are presented in two sections, (1) the key characteristics of the process of HIV service establishment and scale-up in study sites and (2) analysis of the influence of this process on administrative and social accountability within and across the sites. We use the terms ‘health worker’ and ‘provider’ interchangeably to refer to any individual employed by the Ministry of Health and working in a public health facility.

### Key characteristics of HIV service establishment and scale-up

Table 1 outlines key characteristics of the four health centres. In each centre HIV services were established as stand-alone ‘ART clinics’; this occurred during 2006 in Health Centre 1 (HC1), 2009 in Health Centre 2 (HC2), 2005 in Health Centre 3 (HC3) and 2007 in Health Centre 4 (HC4). While ART clinics were located within existing health centre grounds, almost all material and technical support was initially delivered by PEPFAR-funded non-government implementing partners.

**Table 1:** **Health centre demographic information & features of HIV service scale up**

Demographic features	Health centre 1	Health centre 2	Health centre 3	Health centre 4
Designation	Urban	Rural	Urban	Peri-Urban
Official catchment population*	62,579	15,000	101,972	43,850
Official opening hours*	Day: 8:00–17:00	Day: 8:00–17:00	Day: 8:00–17:00	Day: 8:00–17:00
	Night: 17.30–7.30	Night: 17.30–7.30	Night: 17.30–7.30	Night: 17.30–7.30
Service departments**	OPD, MCH,TB, ART, LAB, EH	OPD, MCH,TB, ART, IPD, LAB, LABOUR, EH	OPD, MCH,TB, ART, LAB, EH	OPD, MCH,TB, ART, IPD, LAB, LABOUR, EH
Professional staff*	41	5	46	22
Lay staff*^	29	5	46	12
Common features of ART clinic establishment (c. 2005–2008)	• New stand-alone building for ART clinic in three sites (HC1, HC3, HC4)			
	• Externally funded/supported supply chain & laboratory services			
	• Recruitment & training of adult & peadiatric peer educators/Establishment of peer support groups			
	• NGO funded/run in-service training for select professional staff			
	• Donor-funded ‘overtime’ payments for professional staff working in the ART clinics			
	• NGO supported quality assurance systems			
	• Electronic medical records in three sites (HC1, HC3, HC4); ART specific stationary at all sites.			
Common features of ART clinic scale-up & transition (c. 2009–2011)	• Removal of donor-funded overtime payments			
	• Scale up of MoH-run HIV in-service training for all professional staff			
	• Formal inclusion of ART clinic services in routine duties of all professional staff			
	• Scale-back in NGO support for lay personnel (including peer educators & defaulter tracing)			
	• Scale-back in NGO support for quality assurance programs			
	• Externally funded but MOH managed ART supply chain			
Common effects of ART clinic on facility operations & relationships	• Improved infrastructure & technical capacity to deliver ART.			
	• Early improvements in HCW motivation and clinical standards in ART department.			
	• Lay personnel enabled efficient administrative & non-clinical functions in ART department.			
	• Early intra-cadre jealousies around opportunities for HIV training, overtime payments and better work conditions in ART clinics.			
	• Additional fragmentation (stand-alone ART clinics) of health centre management & operations.			
	• Strong perception amongst providers that HIV services were exceptional to their core duties (especially HC1, HC2, HC3).			
	• Perceptions that HIV services constituted additional/over work, undermining staff morale and service values.			
Particular effects of ART clinic on facility operations & relationships	Enduring intra-cadre jealousies around overtime payments & superior work conditions in stand-alone ART clinic continued to undermine provider cooperation & continuity of care between ART clinic and other departments.	Small cadre of professional staff frequently overwhelmed by requirements of additional HIV services. Effects exacerbated by weaker supervision & quality assurance afforded to rural (as opposed to urban) sites.	Very large patient numbers & decreasing NGO support for lay personnel in scale-up phase led to marked decline in administrative functionality (e.g. missing files; queue bunching) and frequent patient-provider confrontations.	Overall in-charge able to use early gains in performance standards & staff morale in ART clinic to strengthen overall operations via whole-of-clinic meetings/integrated OPD/ART service delivery as levers.

*At the time of study.

**OPD = Outpatient Department; MCH = Maternal and Child Health department; TB = Tuberculosis treatment department; ART = antiretroviral therapy clinic; LAB = laboratory; EH = Environmental Health department; IPD = Inpatient Department; LABOUR = labour ward.

^Includes paid or stipendiary lay staff with a formal terms of reference; does not include *ad hoc* voluntary lay staff.

As summarised in Table 1, key components of the establishment phase of ART clinics included: training and appointment of a new departmental ‘ART in-charge’ within each facility (all sites); substantial investment in new or renovated infrastructure (HC1, HC3, HC4); clinical training for select staff (all sites); recruitment of lay health workers to carry out essential non-clinical duties including patient education and information dissemination (all sites); establishment of an HIV-specific electronic medical record system (HC1, HC3, HC4); a HIV-specific supply chain for antiretroviral drugs and other commodities (all sites); and the provision of non-government partner-assisted quality assurance and quality improvement support (all sites). Additionally, in order to overcome chronic staff shortages, in the three urban health centres (HC1, HC3, HC4) ‘overtime’ payments were provided to existing healthcare workers to work extra shifts in the new ART clinics. In-service training in HIV medicine was paid for and delivered by non-government implementing partners with accreditation provided by the Ministry of Health. Critically, however, early involvement by District Health Management Teams in HIV service implementation or oversight remained limited [25].

The scale-up and transition phase saw some shifts in activities and support for HIV services. In three facilities, in-service training for professional staff was extended to include more nurses and clinical officers (HC1, HC3, HC4). However, cuts in funding for direct salary support meant that HC2 and HC3 both lost lay registry and counselling staff. This period also saw a removal of the donor-funded overtime payments in HC1, HC3 and HC4 and, in an effort to move responsibility for oversight to District and Provincial offices, a scale-back of the quality assurance programs run by non-government implementing partners. At the time of study, quality assurance visits to all four sites had become increasingly *ad hoc*, compared to the nearly weekly visits that had taken place between 2006 and 2009.

### Administrative accountability

Mechanisms of administrative answerability and enforceability should help to make transparent and justify the nature of individual or team performance as well as enable rewards or sanctions for that performance. In Zambian primary health centres, as outlined in Figure 2, mechanisms of administrative answerability included documentation of service activities in medical records, hard-copy registers and tally sheets, production of summary activity reports and performance review and feedback by District administrators. Mechanisms of administrative enforceability were more limited, but included public sector disciplinary processes and horizontal staff transfers as well as the work norms that shaped health worker attitudes and work patterns.

#### Initial improvements in administrative answerability in the ART clinics

Administrative answerability in the ART clinics of all four health centres was initially strengthened via the considerable investment in health system ‘hardware’ as described in the previous section. Improved infrastructure, guaranteed drugs and other medical commodities, training for professional staff and the recruitment of new lay personnel all ensured the clinics had material capabilities necessary to deliver HIV care and treatment according to Ministry of Health issued guidelines.

*With ART we received the big building; and supplies and drugs. We had support. Even the Peer Educators [were recruited and introduced].* Nurse, HC1.

**Figure 3:**
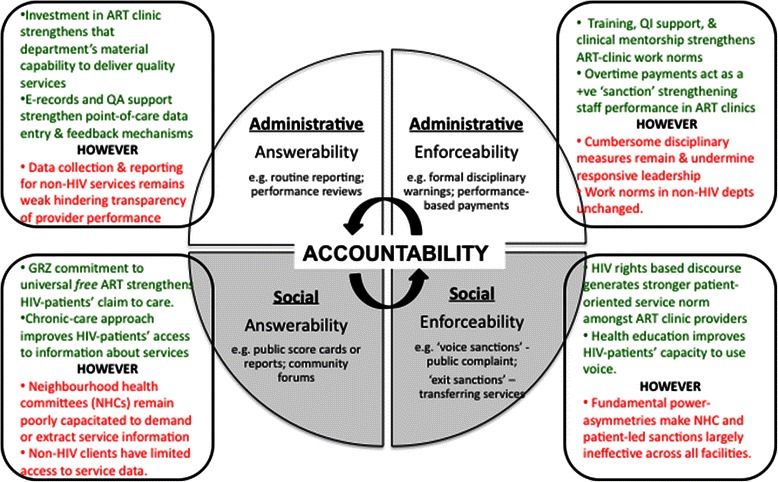
**Impact of HIV scale-up on mechanisms of administrative and social accountability.**

Just as importantly, providers’ ability to collect and disseminate information to demonstrate strong performance was improved (Figure 3). Introduction of a more rigorous HIV health information system, including in three sites (HC1, HC3, HC4) an electronic medical record system, facilitated more accurate and timely documentation of patient and service data. This in turn was used in quality assurance reports and performance reviews, making routine assessment easier and more transparent.

#### Initial improvements in (internal) enforceability in the ART clinics

During the establishment phase, work norms (internal mechanisms of administrative enforceability) were also improved amongst the teams of professional staff working in the ART clinics. Professional development in the form of health worker training and workshops, in combination with NGO supervision and quality assurance support for HIV services, had a demonstrable effect on providers’ attitudes and service motivation.

*Traditionally, [health workers] had the knowledge they accrued in school, but that was it. They started working and then there were no updates. The morale was down and the culture was to do what you always do, whether it’s right or wrong […]. But when the ART clinics were introduced, we saw updates coming in, in-house meetings, and clinical meetings. And that changed the culture quite a lot. The older culture was: “I know I do this, I will do it this way because I have been doing it this way forever.” So change was very hard. But with ART clinics came support and mentorship. And also there was that exchange of skills. It had a huge impact.* In-Charge, HC4.

In the three urban/peri-urban sites (HC1, HC3, HC4), overtime payments were also described as a powerful, albeit short-term, mechanism of enforceability, as a material incentive as well as a more symbolic form of motivation.

*Receiving those payments I felt that I was being appreciated for doing my job well. We were given what we needed, and we were supported.* Nurse, HC4.

*In the ART clinics they have been [paid and therefore] motivated. So there is teamwork and they care for the patients;* In-Charge, HC1.

### Weakening of mechanisms of accountability in the ART clinics over time

Early gains in administrative answerability and enforceability in the four ART clinics were not sustained over time. Key structural factors contributing to increasing laxity in ART clinic performance included increasing patient numbers (without comparable increases in health care workers) and a decline in direct salary support for some lay personnel (e.g. peer educators and data entry clerks) during the transition phase. These changes were related to macro-level decisions regarding donor funding rather than micro-level processes. As part of a broader program of transition, for example, implementing partners were required to cut back on direct support for various lay cadres including data associates, registry clerks and peer educators. Finances for these cadres were re-directed through Provincial offices, but re-hire was delayed for various reasons including lack of Ministry-approved ‘establishment’ positions. At the clinic level, reductions in the number of filing clerks, peers and data associates had significant ramifications for ART service quality, interacting with broader quality determinants (e.g. weak supervision and chronic staff shortages) to affect almost every step of the care pathway including:

- Longer registration queues due to lack of staff to find paper medical records.
- Reduction in the time dedicated by peer educators to group patient education and individual adherence counselling.
- Insufficient support staff to transport files around the clinic, resulting in bottlenecks and lost files.
- Large backlogs of paper files awaiting electronic data entry – creating storage, filing and queue management problems as well as data quality problems.
- Long queues increasing tension amongst patients and encouraging clinicians to take shortcuts to ‘clear the queue’.

The cessation of ART overtime payments during the transition period was another key factor, effectively removing the only formal mechanism of enforceability associated with ART service performance. Removal of overtime payments also revealed a now established perception among health workers that HIV services were *additional* to, not a part of, their core duties, making the new MOH policy stipulating HIV service delivery be part of routine duties difficult to enforce.

*Now that motivation [payment] has been taken away why should I work in the ART clinic? It is extra. No, I will just do my job*. Clinician, HC3.

*I used to work in the HIV department. But now, I can’t. There is unfairness in the working hours. I was told to be working long hours [in the ART clinic] with no incentive. So I tabled it with the sister in-charge and asked for a departmental transfer.* Nurse, HC1.

In two sites (HC1, HC3), overall in-charges even reported instances of staff boycotting the ART clinic as a result of this policy shift.

*Now, because there are no payments, [the staff] from ART clinic are saying: “No, I can’t even work. You want me to work for free!” So now it is different.* Overall In-Charge, HC1.

### Limited gains for administrative accountability in the wider health centres

As can be seen in Figure 3, investment in HIV services also had comparatively few positive flow-on effects in non-HIV departments. Mechanisms of administrative answerability in the broader health centres, including summary service reports and performance reviews in non-HIV departments, generally remained unchanged by the establishment of the ART clinics. Nor did the new services have a positive effect on mechanisms of enforceability in the wider health centres, with the sanctions available to in-charges (e.g. formal disciplinary action or intra and inter clinic transfers) still cumbersome and largely ineffective.

Provider work norms in non-HIV departments were also unchanged by the nascent cultural shift taking place in the ART clinics. In HC1, HC3 and HC4, tensions around the inequitable service conditions of providers working in ART clinics appeared to exacerbate the apathy and disillusion with which many other providers went about their work. This had particular ramifications for internal referrals and continuity of care.

*They used to say of us [ART staff]: “you are highly paid, highly looked after”. They looked at the environment in which we were working in the ART clinic and said we were better off.* Nurse, HC1.

*That tension influenced our work because we saw [staff members] saying: “as long as you are a HIV patient, we [in OPD] will not attend you. Let those other health workers [in the ART clinic] attend to you, since they are trained and paid extra.”* Clinician, HC3.

Nor did investment in establishing the ART clinics improve the strategic leadership capacity of departmental and overall in-charges. Rather, the stand alone nature of the ART clinics, complete with external supervision and NGO-led quality assurance mechanisms, resulted in several in-charges explaining how they had paid little attention to the ART clinics during the establishment period precisely because they were so well resourced.

*There was so much support for training and supplies in the ART clinic. They never complained because they had everything they needed. So I could just leave it and concentrate on other things.* Overall In-Charge HC1.

Weak management of the mostly negative responses to the removal of ART overtime shifts (e.g. boycotting of the ART clinics) was one example of in-charges’ generally weak leadership capacity in this regard. Weak or absent management of other endemic human resource management problems include absenteeism and moonlighting was also evident in several in-charges’ fatalistic approach to these issues.

*They [the staff] just have their own agenda. There is nothing I can do*; Overall In-Charge, HC3).

The overall in-charge in HC4 was an exception to this general finding. Presiding over the integration of an initially stand-alone ART clinic with the outpatient department, he described some clear improvements in facility-wide administrative accountability. Specifically, he was able to use the appointment of a single departmental in-charge for the newly merged OPD and ART departments, and the consolidation of the formerly separate staff rosters, to help reduce unexplained staff absenteeism arising from clinicians and nurses switching between the two departments.

*When we integrated, we were able to cut out on that shift switching whereby some senior staff were really taking advantage.* Overall In-Charge, HC4.

Integration also reportedly helped the overall in-charge to improve expectations for the quality of general outpatient services through the use of ART standard operating procedures. The routine measurement and recording of patient vital signs and provision of patient education, for example, were re-introduced for OPD clients following service integration. Notably, although HC2 and HC3 had also recently integrated OPD and HIV departments, they retained separate OPD and ART in-charges and few of these benefits were documented.

### Social accountability

In a health service setting, effective mechanisms of social answerability should enable clients to understand and assess service availability, quality and responsiveness. By enabling data collection, dissemination and feedback, mechanisms of social answerability serve to ensure transparency of service availability and help to justify service quality. Mechanisms of social *enforceability,* meanwhile, include ‘voice’ and ‘exit’ sanctions [43]. Voice sanctions may encompass written or verbal demands made to and about service providers in one-on-one or group settings, public forums, or via public and social media. In the context of health service delivery, exit sanctions refer to patients’ capacity to ‘vote with their feet’ by attending a different facility. Voice and exit sanctions do not automatically equate with enforceability, however. They are examples of mechanisms that may either partially or wholly compel a stakeholder to act or make a change. The efficacy of such mechanisms is contingent on the way they are enacted (e.g. collective or communal ‘voicing’ of a demand versus individual complaints) as well as a range of structural conditions including legislative environment and/or informal or formal power relations [45].

### Limited strengthening of mechanisms of social answerability

In Zambian primary health centres neighbourhood health committees (NHCs) constitute an important mechanism of social answerability *and* enforceability (Figure 2). Elected or appointed to ensure citizen representation for each facility’s catchment population, NHCs are made up of 8–12 ‘zone chairpersons’ who meet monthly at the health centre, and who preside over smaller zonal committees in between times. The *de jure* purpose of the NHCs is to ensure answerability through information exchange and to enable joint decision-making between providers and the community in relation to service priorities. However NHC committee members in all four facilities in this study listed their most important duty as supporting and supplementing health centre services (e.g. child growth clinics, vaccination drives, outreach programs or health information campaigns), with little evidence of capacity or opportunity to hold health centre staff accountable.

*Our job, it is to support the clinic [services]. Like during under-five activities or if they are doing immunizations in the community. Then we support them and also tell people about these services.* NCH Chair, HC1.

Various factors underpinned the NHC focus on assisting with service delivery versus ensuring the answerability of health workers. Lack of human resource capacity within the health centres meant that professional staff placed significant pressure on committee members to provide stop-gap measures for service delivery.

*We really depend on those guys [the NHC]. I don’t know how we would do our environmental health outreach and MCH activities because we lack staff. Even with these volunteers we still lack capacity.* EHT, HC4.

More fundamentally, NHC members appeared to lack information that would enable them to compare *de jure* health policies with *de facto* services. Nor did NHCs receive any support or training to develop the sorts of communication and advocacy skills necessary to enforce any breaches in performance.

The standing appointment of the health centre in-charge to the position of NHC secretary was also important. Although rationalized in terms of ensuring staff representation at every NHC meeting, the appointment weakened committees’ capacity to act as an independent adjudicator of facility performance, make demands for information or issue complaints in relation to service responsiveness. In two of the health centres (HC1, HC2) the overall in-charges were also responsible for NHC meeting minutes, wielding significant power over the recording and representation of issues at future forums.

Critically, the establishment and scale-up of ART clinics in relative isolation from mainstream clinic operations in these four sites did little to strengthen the capacity or orientation of NHC members to invoke their nominal authority as a mechanism of social accountability (Figure 3). Nor did HIV scale-up activities reinforce the implementation of other mechanisms of answerability, such as the Ministry-mandated ‘patient feedback’ boxes. Health centre audits at the time of study (Jun. – Nov. 2011) found only one of the four facilities (HC 1) with an observable feedback system, though this was described by several staff as rarely used.

### The mixed impact of HIV scale-up on internal mechanisms of enforceability

Some positive effects of HIV scale-up on aspects of social accountability were nevertheless identified. Interviews with health workers demonstrated that HIV service scale-up resulted in a substantial shift in the attitude of professional staff towards HIV-patients. Providers in all four sites clearly articulated the needs of HIV patients as ‘*special*’ or *‘different’,* commenting on the need for empathetic and high quality care when dealing with this group. Some described the intensive material investment in the ART clinics as evidence, in-and-of-itself, of the special nature of this population. These shifts in attitude strengthened work norms related to HIV services, a critical internal mechanism of enforceability.

Paradoxically, however, this shift in attitude towards HIV-patients seemed to exacerbate the perception held by some providers that *non-HIV* outpatient clients were less in need and less deserving of responsive and ethically informed care.

*These OPD patients, they are not patients. Generally speaking twenty five percent in a day are genuine cases… seventy five percent are not genuine cases […] they are malingerers.* Clinician HC1.

Interviewer: *Why do OPD patients carry their own file around the clinic, while HIV patients have their files delivered to the clinician by a staff member?*

Clinician HC3: *With the HIV files, you need to be careful of confidentiality*

Interviewer: *Shouldn’t OPD files also be confidential?*

Clinician HC3: *With the OPD files, it’s just like malaria and cough. So with these guys we don’t usually encourage confidentiality. There’s no stigma for OPD patients, but with HIV more care is needed.*

### Incidental impact on external mechanisms of enforceability

As summarised in Figure 3, providers in all four sites reported that HIV service scale-up strengthened HIV-patients’ willingness to make demands about aspects of their treatment or to request transfers. Providers attributed this shift to the chronic-care approach of the ART clinics, which included routine counselling, health education talks and a standardized approach to service-delivery that helped HIV-patients understand both their disease and the processes involved in their treatment^d^.

*In the HIV department there is routine. There is order. And the patients, they know what to expect and then they can demand it.* Lay Counsellor, HC2.

*For ART patients it’s standard, it is being done for every visit that the patient comes. So the patient is going to be educated to say: “When I arrive at the clinic I’m going to listen to a health talk, I’ll have my vital signs checked or maybe I’ll go for adherence, etc., etc., because that is a standard and it is being done.” That sinks in the patients’ mind and they expect that to be done. But on the other side [in OPD] things are not being done properly; there is no “system” if I may call it.* Nurse, NGO Partner.

Both observation and interview data confirmed that HIV-patients' improved understanding of their treatment and service environment improved their capacity to use 'voice' sanctions as they were more confident to demand certain services (e.g. measurement and recording of vital signs) and point out or demand redress for what they saw as service breakdown (e.g. loss of paper medical files). Enforceability against such demands, however, remained more *ad hoc*.

### Overall production of social accountability generally not improved

Although these outcomes were positive, providers’ attitudinal shifts and HIV patients’ improved awareness and health literacy did not substantively improve social accountability overall within the health centres. Lack of investment in structures and processes that would enable and strengthen collective action (such as the NHCs) meant that strengthened individual capacity to ‘voice’ complaints did not translate into effective mechanisms of enforceability. Providers’ ongoing perceptions of being overworked, underpaid and unable to influence the macro-level factors shaping their work environment, moreover, continued to be influential in shaping their service patterns and work norms [24]. Staff in several facilities (HC3, HC4), for example, expressed a sense of frustration (rather than pride) when describing patients who transferred to their facility, even when it implied superior performance.

*There are too many patients. There is too much work. I can’t stop [the patients] but they should just [go elsewhere].* ART Clinic Nurse, HC3.

*The [patients] who come from other catchment areas should just stay within. They shouldn’t come here. We can’t cope.* ART In-Charge, HC1.

Thus, neither the shift in providers’ internal norms vis-à-vis the entitlements of HIV patients, nor the improvements in HIV patients’ capacity to ‘voice’ their needs, was sufficient to improve social enforceability in the health centres overall. At the time of study, moreover, providers in the ART clinics displayed similar work patterns to those in other departments, including frequent absenteeism and a ‘queue clearing’ approach to patient care.

## Discussion

This study set out to explore whether, and in what ways, the process of HIV scale-up influenced key drivers of service quality and responsiveness in four Zambian health centres. Specifically, we sought to understand how the introduction of HIV services influenced the dual domains of administrative and social accountability, via mechanisms of answerability and enforceability.

Previous work on the impact of HIV care and treatment scale-up on frontline services in Zambia by Brugha *et al.* pointed to early improvements in the availability of ART but mixed findings with respect to improvements in the availability and uptake of other (non-HIV) services. Critically, the authors point to the need for more explanatory research to “*move beyond correlation studies to analyse the processes within facilities to understand and explain such trends*” [17].

The findings presented in this paper go some way to address such research gaps, demonstrating that the early, resource-intensive investment in the ART clinics contributed initially to strong *administrative* answerability within the ART clinics at all four sites by helping to establish the material capabilities necessary to deliver and monitor service delivery. Simultaneous investment in system ‘software’ via external supervision and professional development helped to promote transparency around individual and team performance. In combination with improved data collection and routine monitoring, this investment in system software also had positive effects on administrative *enforceability* in the ART clinics, through strengthened provider motivation and work norms, if not through any overt improvement in clinic managers’ disciplinary capacity.

Well-resourced ART clinics and the implementation of a chronic care service model had an incidental albeit important impact on *social* accountability in the ART clinics. A heightened sense of responsibility for delivering high quality care to HIV patients was evident among providers in all four facilities, and was linked to providers’ sense of empowerment arising from their well-resourced service environments and the perception that HIV-patients were in particular need of care. Such findings highlight the way normative behaviour – a critical internal mechanism of accountability – may be strengthened via dynamic interactions between adequate resourcing and robust data systems on the one hand, and strong leadership focused on relational aspects (notably trust and communication) of service delivery, on the other.

Notwithstanding these gains, establishment and scale-up of HIV services had relatively few positive impacts on the production of accountability in the wider health centres. Mechanisms of administrative accountability, including data collection and performance review mechanisms in other departments were largely unchanged by the HIV scale-up processes. Nor did these activities strengthen the capacity of in-charges to provide strategic leadership or enforce performance standards, despite placing pressure on these individuals to manage an increasingly complex logistical and human resource landscape. In-charges generally weak handling of the tensions surrounding the introduction, and subsequent removal, of ART overtime payments was one example and observations from other settings, including South Africa, have flagged the need for more explicit investment in leadership and stewardship to ensure sustained improvements in quality and responsiveness of both HIV and other primary-level health services [18]. The exceptional example of HC4, meanwhile, provides evidence of how even in generally weak systems, where public sector service values are eroded or latent, strong leadership plays an important role in helping to shift service patterns, work culture and overall performance.

Rifkin [47] argues that social accountability is only truly present when those affected by decisions or actions have some type of effective recourse or process to ensure their needs and concerns are dealt with fairly. In this respect, substantive and long-term gains in social accountability – both in the ART clinics and beyond – were elusive. Despite a positive shift in providers’ attitudes towards HIV-patients, and strengthened capacity of HIV-patients to invoke ‘voice’ sanctions, the scale-up of HIV services contained no specific activities designed to address the deeply rooted information and power asymmetries affecting social answerability and enforceability more broadly. The gap between NHCs’ *de jure* purpose and *de facto* activities, for example, was unchanged by the introduction of HIV services and remained heavily influenced by NHC members’ imperfect understanding of health centre performance standards and their lack of authority in facility planning processes.

In the absence of a more coordinated application of ‘voice’ or ‘exit’ sanctions at the meso- and macro-levels, we also found that the positive but unintended gains in relation to patients’ capacity to ‘voice’ issues had little impact on the deeply rooted norms and macro-level determinants of service quality such as chronic human and other resource shortages.

As Walt [48] notes, health provider behaviour is embedded in the social institutions and power relations of their working environment. Such uneven power dynamics remain an unfortunate but notable feature of many LMICs’ health services [49-52]. Growing experience of programmatic work and a nascent body of research in the area of social accountability highlight potential areas of intervention and system strengthening in this domain [53,54]. Mechanisms such as community dialoguing and patient score-cards, in particular, have been the focus of an increasing number of programs [55,56]. A recent small-scale evaluation of a ‘citizen voice and action’ program in Zambia provided local evidence of the importance of addressing entrenched power dynamics between patients or communities and facility and District level health officers through concerted investment in dialoguing and networking skills on both sides [57].

Influenced by the assumption that front-line or lower-level health services represent simple or even mechanistic systems [15,37,58], service interventions, particularly those planned and financed through global health initiatives, are often based on the logic of linear causal pathways, which assume that investment in one area of a health facility will produce automatic, predictable and positive effects overall. This assumption is in turn underpinned by a failure to recognise the complexity of both the interventions themselves (with their long and multi-factor causal chain) and the people-centredness of the target (micro-level) health systems [59].

One of the implications of failing to engage with the complexity of micro-level health systems is highlighted by our finding that administrative accountability in three of the four ART clinics in this study actually weakened over time. During the period of HIV service establishment, NGO and government implementers focused attention on establishing strong, self-sufficient ART services. With the focus squarely on emergency scale-up, less consideration was given to the interaction that these frontline ART services would ultimately have with broader health centres. Inevitably, as the ART clinics began to experience more structural pressure from increasing patient numbers and as the distinction between ART and general health centre staff and systems became more blurred, the service patterns and work norms that shaped providers’ behaviour in health centres as a whole, started to merge. Structural conditions – including donor policy shifts that resulted in the removal of financial incentives, weak GRZ capacity to meet resource gaps and the poorly implemented transfer of technical support activities – also contributed to a shift of ART service patterns and work norms towards those found in the wider facilities.

Such findings demonstrate how despite some initial gains in service quality and responsiveness in one specific service domain, sustained improvements were ultimately inhibited by the selective nature of the intervention.

## Conclusions

The research presented in this paper adds to a small but important body of work that provides policy-relevant evidence of the complex nature of micro-level health systems and the way targeted service interventions have multiple and often unintended effects on system performance. Our findings highlight the importance of shifting the understanding by global health technocrats, national and sub-national policy makers and implementing officials of the interaction between targeted interventions and the health systems in which they are located. To (at worst) do no harm, and (at best) produce an overall system strengthening effect, policy makers and implementers of disease-specific service interventions must give more specific and strategic consideration to understanding hardware-software interactions and their relative impact on service drivers. This requires a re-orientation of disease-specific interventions away from mechanistic logic-models and towards realistic assessments of the complex, social and adaptive nature of health systems at all levels, and highlights once more the importance of careful planning and adequate investment in both system hardware and software to achieve sustained and quality service-delivery at all levels.

## Endnotes

^a^The transition phase was marked by poor communication between policy-makers, NGO implementing partners, and front-line service providers. Underpinned by the lack of operational planning for what ‘transition’ would mean, lack of information in the health facilities in Zambia, for example, meant that many health workers were either unaware that NGO implementing partners no longer had the funds or the remit to provide technical support, or that these funds had been redirected to the Ministry of Health. A perception of being ‘abandoned’ was noted throughout the data collection period, and similar confusion has been reported elsewhere [24,29].

^b^We acknowledge some conceptual overlap between the domains of accountability described above, and the role of trust in health care settings. For the sake of conceptual clarity, however, our focus in this paper lies exclusively with accountability. Future publications will examine the interactions between the two.

^c^We recognise the importance of trust but for the purposes of analytical clarity choose to address this mechanism in a separate paper.

^d^Although not explored in this study, it also seems likely that the public information dissemination, education and communication campaigns run between 2000 – 2006 in Zambia (see for example [60,61]) may have helped to improved clients’ general health literacy related to HIV care and treatment.
